# Selective orbital reconstruction in tetragonal FeS: A density functional dynamical mean-field theory study

**DOI:** 10.1038/srep46439

**Published:** 2017-04-18

**Authors:** Luis Craco, Stefano Leoni

**Affiliations:** 1 Instituto de Física, Universidade Federal de Mato Grosso, Cuiabá, MT, 78060-900, Brazil; 2 School of Chemistry, Cardiff University, Cardiff, CF10 3AT, UK

## Abstract

Transport properties of tetragonal iron monosulfide, mackinawite, show a range of complex features. Semiconductive behavior and proximity to metallic states with nodal superconductivity mark this *d*-band system as unconventional quantum material. Here, we use the density functional dynamical mean-field theory (DFDMFT) scheme to comprehensively explain why tetragonal FeS shows both semiconducting and metallic responses in contrast to tetragonal FeSe which is a pseudogaped metal above the superconducting transition temperature. Within local-density-approximation plus dynamical mean-field theory (LDA+DMFT) we characterize its paramagnetic insulating and metallic phases, showing the proximity of mackinawite to selective Mott localization. We report the coexistence of pseudogaped and anisotropic Dirac-like electronic dispersion at the border of the Mott transition. These findings announce a new understanding of many-particle physics in quantum materials with coexisting Dirac-fermions and pseudogaped electronic states at low energies. Based on our results we propose that in electron-doped FeS substantial changes would be seen when the metallic regime was tuned towards an electronic state that hosts unconventional superconductivity.

Over the past few years iron-based superconductors^1^,^2^,^3^ and topological insulators (TI)^4^ have attracted great attention in material science and condensed matter physics. In theory, TIs are predicted to be bulk semiconductors with protected surface and edge states^4^. Most TIs are *p*-band materials characterised by strong spin-orbit interaction and linear, Dirac-like band dispersion near the Fermi energy (*E*_*F*_). On the other hand, iron-based superconductors are materials in which the electronic states near *E*_*F*_ are derived from Fe-3*d* orbitals. Given the fact that the spin-orbit interaction on iron is rather weak compared to Bi-based *p*-band TIs might look as if these two systems belonged to a different material class. However, a perusal of extant experimental^5^,^6^,^7^,^8^,^9^,^10^,^11^ and theoretical^12^,^13^,^14^ studies suggests band topology with Dirac cones^15^ in iron-based superconductors. The Dirac cone band dispersions in iron-based superconductors are normally absent in the non-magnetic phase, but surprisingly they appear in the magnetically ordered state^9^,^12^.

In the recent history of Dirac-fermion physics in iron-based superconductors^15^, BaFe_2_As_2_ was the first material to reveal an anisotropic Dirac point near *E*_*F*_^5^. In this 122-system the Dirac cone state appears to be originated from nodes in the spin-density-wave (SDW) gap via zone foldings in bands with different parities^12^. Interestingly, this state coexists with superconductivity in Ru-doped BaFe_2_A_2_ until SDW ordered phase vanishes^7^,^16^. Similar behavior was also observed in Ru-doped LaFeAsO^17^, suggesting that the appearance of Dirac fermions in iron-pnictide superconductors is accompanied by SDW instabilities. However, recent theoretical studies indicate that iron-chalcogenide superconductors without SDW instabilities can carry nontrivial topological properties^14^. Particularly interesting is the single layer FeSe grown on SrTiO_3_ substrate where nontrivial (*Z*_2_) topology can be tuned through band inversion at *M* point of the Brillouin zone^18^. Moreover, coexistence of Dirac fermions and superconductivity is also seen in iron-chalcogenide solid solutions with suppressed SDW long-range order^10^. Taken together, these findings might be an evidence that the origin of the Dirac cone state in iron-chalcogenides is different from that seen in iron-pnictides^11^. Thus, it is important to explore the physical mechanism which generates such a topological anomaly in the non-magnetic electronic structure of layered iron-chalcogendes. With this caveats in mind, in this work we reveal a novel aspect of Dirac fermion physics in iron-chalcogenides which is intrinsically tied with their correlated multi-band nature. Using tetragonal iron monosulfide (FeS) as a suitable template we show its orbital-selective electronic reconstruction at the border of the first-order Mott-Hubbard metal-insulator transition point.

Tetragonal FeS is found naturally as the mineral mackinawite, which was identified as the initial product from corrosion of metallic iron on sulphur^19^. It is also the first solid phase to be precipitated during iron sulfide mineralization mediated by sulfate-reducing bacteria^20^,^21^. Mackinawite influences the bioavailability of environmentally important trace elements^22^ and it seems to play an important role in the iron-sulfur hypothesis on the origin of life^23^. Mackinawite is also of fundamental importance in view of understanding its semiconductive nature^24^,^25^,^26^ as well as the possibility of tuning metallicity and unconventional superconductivity in hydrothermally synthesized samples^27^,^28^. In fact, recent experimental studies show clear evidences for non-metallic behavior in tetragonal FeS at ambient pressures^24^,^25^,^26^. The resistivity of mackinawite remains insulating-like under moderate pressures up to 1.178 GPa^24^ but it might turn metallic at higher pressures for temperatures above 75 K^26^. Moreover, neutron diffraction and Mössbauer studies show no evidence of magnetic ordering down to 1.7 K^29^. Interestingly, mackinawite host magnetic properties which are similar to Fe-based superconductors, particularly those of FeSe_0.88_^30^. This compound exhibits similar temperature-independent Mössbauer spectra down to 4.2 K as in tetragonal FeS. Thus, mackinawite is an ideal system to explore novel phases of quantum matter in correlated electron systems. Indeed, unconventional superconductivity (*T*_*c*_ ≈ 5 K) in tetrahedral FeS single crystals were recently reported by different groups^27^,^28^,^31^,^32^. Similar to tetragonal FeSe superconductor^33^, low-temperature heat transport measurements suggest nodal superconductivity gap in metallic FeS^32^. Interestingly, this new class of iron-based superconducting materials (which are prepared under hydrothermal reactions^28^,^31^) exhibits paramagnetic behavior with different residual resistivity values^27^,^31^,^32^ and large effective mass anisotropy^31^. It is worth noting that sample-to-sample variations were also observed in *μSR* data^34^, but the superconducting behavior seems to be insensitive to the presence of small, non-superconducting magnetic phase in the sample, which could be used in future studies as a probe of the robustness of Dirac fermions against disorder^35^. Additional *μSR* measurements indicate coexistence of low-moment magnetism and bulk superconductivity^36^, where the superconducting transition temperature of superconducting FeS continuously decreases for hydrostatic pressures up to 2.2 GPa. This in turn suggests a pressure-induced high- to low-spin state transition^37^ accompanied by strong Fermi surface reconstruction of bulk t states^38^ in FeS superconductor.

The localized nature of hexagonal FeS (troilite) and its correlated electronic structure has been investigated by photoemission (PES) and inverse-photoemission spectroscopy experiments^39^. Based on this seminal study it is known that the Fe 3*d* bandwidth in PES spectra is 25-to-30% narrower than that predicted by first-principles band-structure calculations. The one-particle density-of-states (DOS) was shown to be accompanied by an intense tail at high binding energies, consistent with the correlated fingerprints found in iron-superconductors^40^. Cluster-model calculations in Ref. fujimori indicate Hubbard-like satellite structures at high energies for realistic values of the on-site Coulomb interaction between 4.0 eV-to-5.0 eV. By introducing self-energy corrections to the band DOS predicted by first-principles band-structure calculations the observed Fe 3*d* band narrowing at low energies and the satellite features at high-binding energies can be explained^39^. However, in spite of these results for troilite the correlated nature of mackinawite has not been explored so far.

Currently, the theoretical understanding of tetragonal FeS is restricted to one-electron band structure calculations^41^,^42^. Specifically, *ab initio* density functional calculations for Fe*X (X* = S, Se, Te) demonstrate that the chalcogen *p* states lie well below *E*_*F*_ and are weakly hybridized with Fe 3*d* states^41^. Hence, the general consensus now is that the most relevant electronic states near the Fermi energy derive from Fe^2+^ (with *d*^6^ electronic configuration) layers with almost direct Fe-Fe hopping. In previous work we undertook systematic local density approximation plus dynamical-mean-field-theory (LDA+DMFT)^43^ studies of iron-based superconductors, showing how bad-metallicity^40^ and doping-induced localization-delocalization transition can be understood within a single theoretical picture. Good semiquantitative agreement with extant experimental data of tetragonal Fe(Se, Te) systems^40^,^44^ serves as support to explore intrinsic, dynamical correlation effects in mackinawite.

A proper microscopic description of localization-delocalization transition^26^,^45^ and the possibility of finding massive Dirac-fermions^11^ in 11 iron-chalcogenide systems is therefore important for understanding the role played by dynamical correlations in the low-energy electronic states of iron-based superconductors. In this work we provide new insights to the problem of multi-orbital (MO) electron-electron interactions in tetragonal FeS, revealing the emergence of an orbital-blocked phase^46^ with linear spectrum and its possible implications for Kondo-like mass enhancement of Dirac fermions in iron-based superconductors^8^.

## Results and Discussion

**Figure 1:**
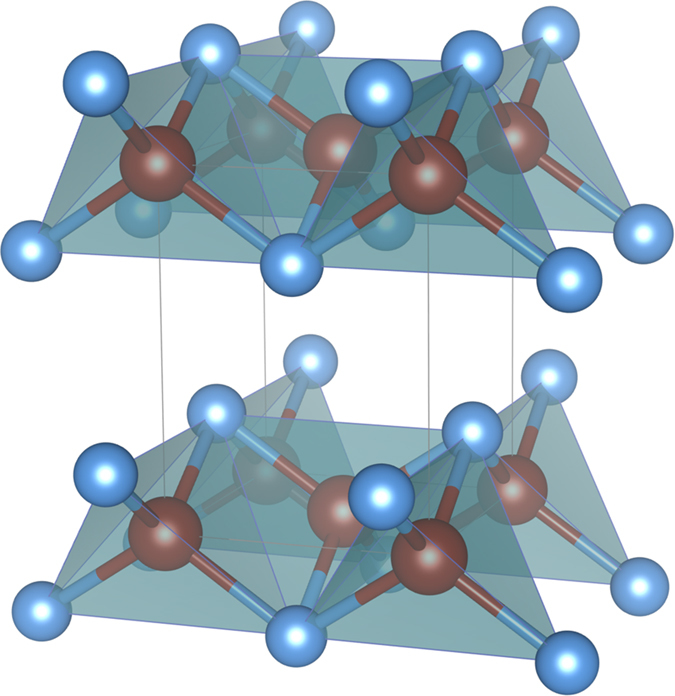
Crystal structure of tetragonal FeS. Large and small spheres represent sulphur and iron atoms, respectively. The origin of the unit cell is chosen at Fe positions. Two layers of edge-connected FeS_4/4_ tetrahedra are shown.

**Figure 2:**
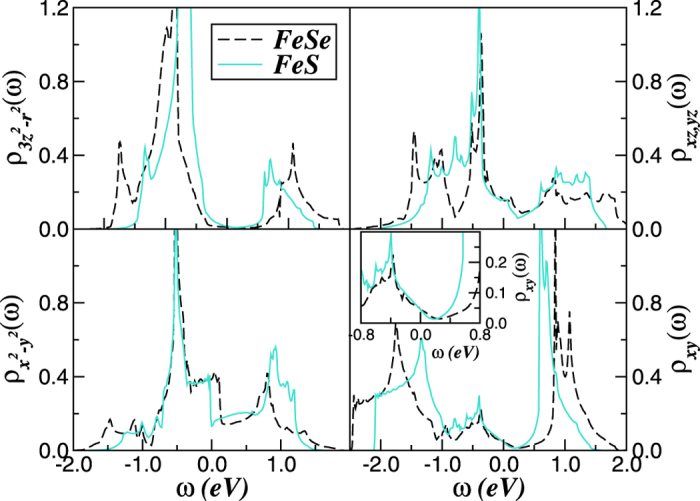
Orbital-resolved LDA density-of-states (DOS) for the Fe *d*-orbitals of tetragonal Fe-chalcogenides computed using the LMTO method: FeSe (long-dashed line) and FeS (solid line). An important feature to be seen is the band narrowing of the LDA DOS in FeS compared to FeSe superconductor. It is noteworthy that all *d*-bands span over the Fermi level. This confirms that the electronic states relevant to Fe-chalcogenides are Fe *d*-states^41^.

Within the tetragonal (space group: *P*4/*nmm*) structure (see Fig. 1), and using lattice parameters taken from experimental data^41^, one-electron band structure calculations based on local-density-approximation (LDA) were performed for the parent compound FeS using the linear muffin-tin orbitals (LMTO) scheme^47^,^48^. Consistent with previous calculations^41^,^42^ our LDA results in Fig. 2 confirms that the active electronic states in mackinawite involve Fe 3*d* carriers. To clarify the underling differences in the bare electronic structure between FeS and FeSe parent compounds, in this figure we also display the orbital-resolved LDA DOS of tetragonal FeSe^44^. The chalcogen-induced anisotropies in the LDA band structure are clearly manifested in our results. An overall reduction of the one-particle bandwidth (*W*) is seen in the orbital-resolved DOS of FeS. While the *x*^2^ − *y*^2^ and *xz, yz* orbitals show less pronounced effects due to smaller ionic radii of sulphur compared to selenium, the 3*z*^2^ − *r*^2^ and the *xy* channels display appreciable one-particle band narrowing and small changes in the bonding/antibonding splitting of the electronic states close to *E*_*F*_, characteristic of the tetragonal unit cell. As seen in the inset (right-lower panel) of Fig. 2 the *xy* orbital shows tendency towards the formation of a *V*-shaped electronic spectrum, which is the one-particle seed towards a reconstructed Dirac-like linear band dispersion in tetragonal FeS, as shown below. As comprehesivelly described in this work, due to one-particle band narrowing correlations effects can increase dramatically in FeS compared to FeSe, leading to an orbital blocking mechanism and appearance of *marginal* Dirac fermions in the *d*-band electronic states of FeS.

Although LDA provides reliable structural information at one-electron level, it generically fails to capture the ubiquitous dynamical correlations in *d*-band compounds, and so cannot access normal state incoherence and Mott-Hubbard insulating states. Combining LDA with DMFT is the state-of-the-art prescription for overcoming this problem^43^. Here, the relevant inputs to our LDA+DMFT treatment for mackinawite are the LDA DOS for the five 3*d* orbitals shown in Fig. 2, the on-site Coulomb interaction *U*, the inter-orbital term *U*′ = *U* − 2*J*_*H*_, and the Hund’s coupling *J*_*H*_. The correlated many-body Hamiltonian for FeS reads


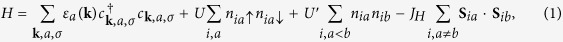


where *a* = (*x*^2^ − *y*^2^, 3*z*^2^ − *r*^2^, *xz, yz, xy*) denotes the diagonalized 3*d* orbitals of FeS and *ε*_*a*_(**k**) is the one-electron band dispersion, which encodes details of the actual one-electron (LDA) band structure. We evaluated the many-particle Green’s functions of the Hamiltonian above within LDA+DMFT^43^, using MO iterated perturbation theory (MO-IPT) as impurity solver^49^. The DMFT solution involves replacing the lattice model by a self-consistently embedded MO-Anderson impurity model, the self-consistency condition requiring the local impurity Green’s function to be equal to the local Green’s function for the lattice. The full set of equations for the MO case can be found in refs 49 and 50, so we do not repeat the equations here.

**Figure 3:**
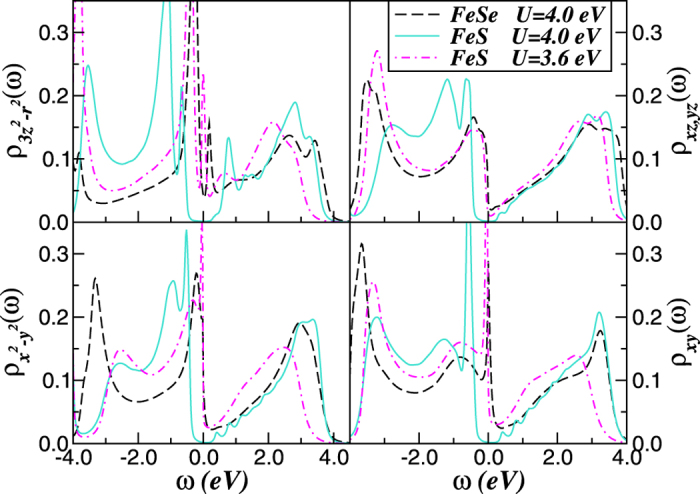
Orbital-resolved LDA+DMFT density-of-states for the Fe *d*-orbitals of tetragonal iron-chalcogenides. FeSe (long-dashed line) and FeS (solid and dot-dashed lines). [The parameters are *U* = 4.0 eV (FeSe) and *U* = 3.6 eV, 4.0 eV (FeS) and fixed *J*_*H*_ = 0.7 eV]. Large spectral weight transfer compared to LDA and incoherent, Hubbard bands at high energies are visible in both systems. Notice the Mott gap at *U* = 4.0 eV for the electronic states of FeS and the good qualitative agreement for the density-of-states obtained with *U* = 3.6 eV and that of FeSe, indicating that FeS might host nodal unconventional superconductivity similar to that of FeSe under suitable perturbations.

We now discuss our LDA+DMFT results. In Fig. 3 we display the effect of electron-electron interactions on the orbital resolved electronic structure of tetragonal FeS. At *U* = 4.0 eV (and *J*_*H*_ = 0.7 eV)^40^,^44^ FeS is a MO Mott-Hubbard insulator with an orbital dependent band gap. Lower- (LHB) and upper- (UHB) Hubbard bands are visible, albeit to a different extent, in all orbital-resolved spectral functions. As seen, the 3*r*^2^ − *z*^2^ orbital shows strong correlation effects with pronounced charge gap and Hubbard bands, while the *x*^2^ − *y*^2^ orbital has less tendency towards local moment formation (LHB). In this figure we also display our previous LDA+DMFT results for FeSe superconductor^44^, to highlight the different orbital blocking mechanism^46^ in these two systems. One striking feature of our results is the good qualitative agreement between the orbital-resolved DOS of FeS for *U* = 3.6 eV and that of stoichiometric *d*^6^ FeSe. Clearly, the two spectral functions show similar behavior at low energies, indicating that FeS is able to host an unconventional superconducting state at low *T* if its electronic structure is properly tuned via suitable sample preparation conditions^27^,^28^,^31^. Our results in Fig. 3 seems to confirm very recent measurements demonstrating that the quasiparticle mass is significantly reduced from that of FeSe in hydro-thermo grown FeS superconductor^51^. Interestingly, this work suggests that metallic FeS is less correlated compared to FeSe, which is consistent with our results showing that the on-site Coulomb interaction *U* in metallic FeS is at least 0.4 eV smaller than that of tetragonal FeSe as in Fig. 3. Taken theory and recent observations on metallic mackinawite together, an increase of itinerancy in nearly ideal tetrahedral single crystals^31^ can be expected, which will in turn reduce the ratio between the on-site Coulomb repulsion and the one-particle bandwidth (*U/W*), driving the electronic states of stoichiometric FeS close to that of tetragonal FeSe superconductor.

**Figure 4:**
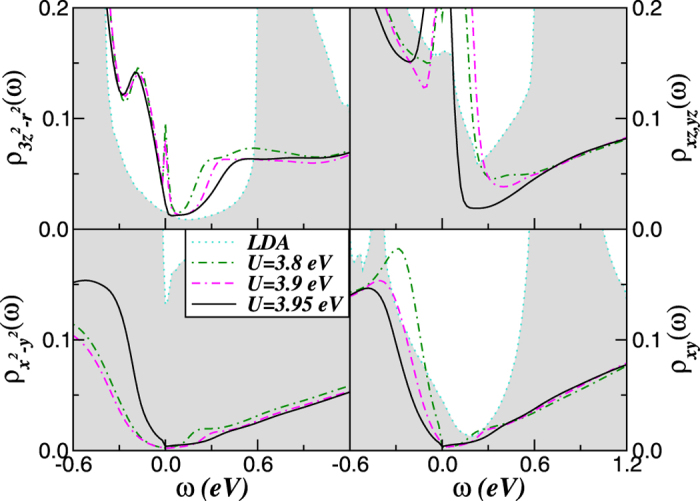
LDA+DMFT orbital-resolved density-of-states of FeS at the border of the correlation driven Mott metal-insulator transition. Notice the modification of the LDA+DMFT spectral functions due to large-scale dynamical spectral weight transfer. While the 3*z*^2^ − *r*^2^, *xz, yz* orbitals remain pseudogaped as in the normal state of FeSe superconductor^44^, an asymmetric *V*-shapped Dirac-like spectrum sets in within the *x*^2^ − *y*^2^ and *xy* channels due Mottness-induced orbital reconstruction. (LDA results are shown for comparison).

To characterize the hidden Dirac-liquid regime^52^ in mackinawite, in Fig. 4 we show its orbital-resolved DOS at the border of the first-order Mott metal-insulator transition point. Fig. 4 shows that coexistence of pseudogapped (3*z*^2^ − *r*^2^, *xz, yz*) and quasi-linear (x^2^ − *y*^*2*^, *xy*) spectral functions near *E*_*F*_ can be tuned in the bulk. Also, the Dirac cones in tetragonal FeS appears to be anisotropic, consistent with observations for BaFe_2_As_2_^5^ whose apex is also located slightly above *E*_*F*_. This result can be taken as an evidence that the interplay between band narrowing and Coulomb perturbations^53^ is a natural way to approach novel multiband effects and Dirac-fermions in tetragonal iron-chalcogenide systems^11^. The critical metal at the border of the Mott transition in FeS should therefore be characterized by pseudogaped electronic (3*z*^2^ − *r*^2^, *xz, yz*) states coexisting with Dirac-like fermions at the x^2^ − y^2^, *xy* orbitals. Thus, within the hidden Dirac-liquid phase the reconstructed Fermi surface is expected to be composed of two distinct components, and future angle-resolved photoemission (ARPES) experiments could verify this aspect.

**Figure 5:**
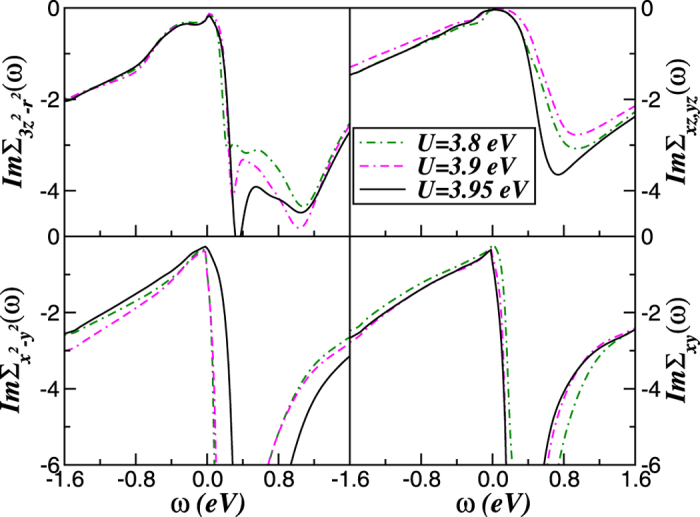
Imaginary parts of the orbital-resolved LDA+DMFT self-energies of tetragonal FeS, showing an orbital-dependent Fermi and non-Fermi liquid behavior. Notice the sub-linear frequency dependence of 
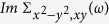
 near *E*_*F*_ and the pronounced particle-hole asymmetry of Σ_*a*_(*ω*) in all *a* = (*x*^2^ − *y*^2^, 3*z*^2^ − *r*^2^, *xz, yz, xy*) orbitals. These results suggest that an exotic metal (comprised of pseudogaped and Dirac-liquid spectrum) as in Fig. 4 can be formed in FeS at the border of the Mott transition.

Our main result in Fig. 4 is that the *V*-shaped liner dispersions with Dirac-cone like carriers^54^ can be tuned by electronic interactions in bulk mackinawite at the border of the Mott metal-insulator transition point. According to our previous results on topological insulators^55^, residual spin-orbit interactions (not included in our theory) may induce topological protection^4^,^15^ and weak antilocalization in tetragonal FeS. Although the weight of the Dirac states is small near *E*_*F*_ and the contribution of the psedogaped bands must be suppressed to extract the unique carrier transport intrinsic to Dirac cones^6^,^7^, the mobility of Dirac-fermions could dominate electronic transport and a linear temperature dependent magnetoresistance as in Fe_1+*y*_Te_1−*x*_Se_*x*_ single crystals^11^ could be observed in metallic FeS. Taken together, the LDA+DMFT electronic structure in Figs 3 and 4 show large-scale changes in spectral weight transfer (SWT) at the critical phase boundary, 3.95 eV < *U*_*c*_ < 4.0 eV. Electron-electron interactions renormalizes the LDA+DMFT results in two steps. First, the MO self-energy [Σ_*a*_(*ω*)] renormalizes the relative band positions depending upon their orbital occupations. The frequency-dependent self-energy (see Fig. 5) causes SWT across large energy scales, drastically modifying the correlated spectral functions. As a consequence of Mottness, the two-fluid metal (comprised of pseudogaped and Dirac-liquid spectrum) found in FeS at the border of the Mott transition is totally disrupted in insulating FeS at normal conditions. As shown in Fig. 5, the relative large range of pure linear spectrum within the *x*^2^ − *y*^2^, *xy* channels in Fig. 4 is closely related to the frequency dependence of the self-energy imaginary parts, which show clear deviation from the *ω*^2^-dependence of a canonical Fermi liquid (FL). Below the Mott transition only the degenerated *xz, yz* orbitals show characteristic FL like features, while the more correlated orbitals display deviations from the −*ω*^2^ FL form at small *ω*, being consistent instead with (sub-) linear *ω*-dependence of *marginal* Fermi liquids^56^. Similar self-energy behavior as in Fig. 5, with sub-liner energy dependence was also found in hexagonal and tetragonal FeSe^57^, suggesting a common scenario of correlation-induced electronic reconstruction in 11 iron-chalcogenides. Interestingly, the departure from the FL behavior caused by many-body interactions has been also reported for quasi-freestanding graphene^58^, showing similar marginal form as found here for the Dirac channels. Motivated by the fact that our orbital-selective linear spectrum is in close proximity to Mottness we dub it as *marginal* Dirac-liquid.

As shown in Fig. 5, the marginal Dirac-liquid state goes hand-in-hand with the development of a *V*-shaped *ω*-dependence in 
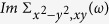
 close to *ω* = 0. This implies that, close to the Mott insulator-metal transition, we have a renormalized (by DMFT) picture where effectively Dirac-like fermions co-exist with incoherent electronic states from the remaining (3*z*^2^ − *r*^2^, *xy, yz*) bands. In our LDA+DMFT, the FL quasiparticle coherence would be destroyed near the Mott transition in the disorder-free case of FeS, as evidenced by the fact that 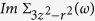 display a sharp kink near *E*_*F*_ while 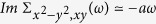 with a pronounced particle-hole asymmetry, implying a vanishing FL quasiparticle residue. Nonetheless, it is possible that, instead of a linear dependence, a weak power-law-in-*ω* dependence could result in experiments: recall that our DMFT self-energies are not inconsistent with a weak power-law (in *ω*) dependence at low energy. In this case, 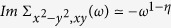. However, similar *V*-shaped spectral functions were also obtained within LDA+DMFT for tetragonal FeSe superconductor^59^, which undergoes an orbital-selective insulating state upon electron doping.

What is the microscopic origin of the orbital-selective features found in the LDA+DMFT solution of tetragonal FeS system near the Mott transition? On general grounds, as *U, U*′ increases a subset of *d*-orbitals gets selectively Mott localized. The metallic phase is then the orbital-selective metal found in various contexts^59^,^60^. Once this selective localization occurs within DMFT, the low energy physical response is governed by strong scattering between the effectively Mott-localized and the renormalized, itinerant components of the matrix spectral function. Within LDA+DMFT for mackinawite, the itinerant fermion spectral function then shows a low-energy pseudogaped form, while the localized spectral function shows a power-law fall-off as a function of energy, as long as the renormalized Fermi energy (*E*_*F*_) is pinned to the renormalized orbital energy of the localized orbital(s). This behavior is understood from the mapping of the corresponding impurity model to that of the “X-ray edge”, where the orthogonality catastrophe destroys FL coherence. The spectral functions then exhibit asymmetric continuum at low energy, instead of symmetric Abrikosov-Suhl Kondo resonance features at low energies, and the metallic phase is non-Fermi liquid. As shown here, the frequency-dependence of the self-energy imaginary parts is important in stabilizing the marginal Dirac liquid state, and consideration of the instability of such an orbital-selective state to a superconducting (SC) state should lead to a strongly frequency-dependent superconducting gap function. (We plan to explore these aspects in a future work on the Fe-chalcogenide family.) As expected, in a correlated electron picture, the physical response functions in the normal state are controlled by both, intrinsic orbital degrees of freedom as well as the *ω* dependent damping originating in the incoherent normal state. Future observation of features such as the presence or absence of an incoherent low-energy peak in X-ray emission spectroscopy (XES)^37^ and the large scale modification of the spectral weight across the electronic transition in metallic mackinawite will provide support to our scenario.

In MO systems the orbital-resolved hopping matrix elements (diagonalized in the LDA) are sensitive functions of orbital orientation in the real crystal structure. Moreover, the 3*d* bands are usually shifted relative to each other because of the action of structural induced changes in the crystal-field splittings: the six *d* electrons found in iron-based superconducting materials are distributed among all *d* orbitals and, in the case of a system with sizable MO interactions, this might induce orbital polarization and SWT. In this situation, MO correlations cause various intimately linked changes: the static MO Hartree shifts, which depends upon the occupations of each orbital as well as on the inter-orbital correlations [*U*′ and *J*_*H*_] renormalizes the on-site energies of each orbital in different ways. In particular, it causes inter-orbital charge transfer between the different *d*-orbitals, self-consistently, modifying their energies and occupations^60^. If the pressure induced high-spin (HS) to low-spin (LS) scenario^37^ is consistent with low-moment magnetism^36^ in tetragonal FeS, the *xy, xz, yz* orbitals in LDA will be lowered in energy while the 3*z*^2^ − *r*^2^, *x*^2^ − *y*^2^ will be pushed further up by the MO-Hartree shifts. The amount of this electronic modification is normally determined by the orbital occupation(s) and by the values of *U, U*′, *J*_*H*_ relative to their respective LDA band width(s), and to the bare crystal-field splittings. As common to correlated electron systems, the dynamical correlations associated with *U, U*′, *J*_*H*_ results in a large-scale dynamical SWT. Here, small changes in the bare (LDA) band structure induced by MO electronic correlations (or by small volume changes^28^) are expected to drive large changes in SWT, drastically modifying LDA lineshapes of mackinawite.

**Figure 6:**
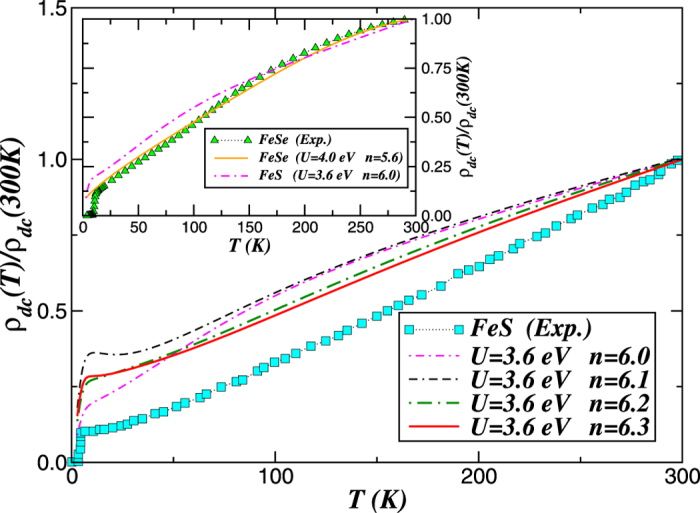
Resistivity versus temperature (normalized to *ρ*_*dc*_(300 *K*)) profiles for pure and doped FeX (X = S, Se) systems. Main panel: Theory-experiment comparison of *dc* resistivity [*ρ*_*dc*_(*T*)] for tetragonal FeS^27^. Notice the quasilinear temperature dependence of transport data above 25 K in experiment, which is qualitatively reproduced by LDA+DMFT for electron doped (6.2 < *n* < 6.3, with *n* being the total occupations of the Fe-3*d* shell) FeS. Compared to experimental data large residual resistivity in theory is found at low temperatures. Inset: Temperature dependence of *dc* resistivities of metallic, tetragonal FeSe and FeS obtained using the LDA+DMFT spectral functions for different on-site Coulomb interaction (*U*) and Fe-3*d.* occupancies (*n*). Notice the qualitative agreement between the LDA+DMFT resistivity for pure FeS and the experimental data of FeSe superconductor^63^, suggesting that stoichiometric FeS might host unconventional superconductivity similar to pseudogaped^62^ FeSe superconductor.

Finally, to elucidate close similarities between the metallic state found for FeS at *U* = 3.6 eV and that of FeSe (see Fig. 4) as well as the crucial role played by MO interactions on electric transport in layered iron-based materials, we show in Fig. 6 the temperature (*T*) dependence of the *dc* resistivity computed using the orbital resolved LDA+DMFT spectral functions, *A*_*a*_(*ε, ω*). Within the Kubo formalism^61^, the *dc*-conductivity can be expressed as


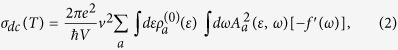


where 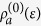 is the LDA DOS of the five 3*d*-bands, *V* is the unit cell volume, and *f(ω*) is the Fermi function.

We now describe our electric transport results for metallic FeS. In Fig. 6, we show the resistivity, 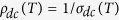, curves of pure and electron-doped FeS (for *U* = 3.6 eV and *J*_*H*_ = 0.7 eV) derived using the LDA+DMFT spectral functions. In the same figure we also display our theory-experiment comparison of the normalized resistivity 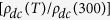 of FeS superconductor^27^. As seen, the LDA+DMFT result for doped FeS (*n* > 6.0) shows the S-like shape characteristic of a pseudogaped metal similar to FeSe superconductor^62^. As expected at low *T*, the FL-like *T*^2^ behavior is not seen in Fig. 6. At commensurate band filling, *n* = 6.0, *ρ*_*dc*_(*T*) becomes more metallic-like at low temperatures, smoothly suppressing the *S*-like form below 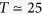 K in theory for *n* = 6.1. At low *T*, 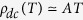 and the FL-like *T*^2^ form is also not observed upon partially electron doping the parent compound. The underlying reason for this behavior is that at small *U, J*_*H*_, the moderately correlated FL behavior cannot be destabilized by a small *J*_*H*_. For large *U* and sizable *J*_*H*_, however, the correlated-Landau Fermi-liquid scale, already driven to small values by sizable *U*, can be readily destroyed by *U*′. As seen, with increasing total electron concentration *n* > 6.0 of the iron *d* shell, *ρ*_*dc*_(*T*) becomes more bad-metallic^59^, smoothly going over to a metalic state with higher residual resistivity at low temperatures. In reality, in tetragonal FeS an unconventional superconducting transition cuts-off this extremely low crossover to a strange metal as seen in Fig. 6. Moreover, in the inset of Fig. 6 we display the normalized resistivities of tetragonal of Fe*X (X* = S, Se) systems, showing similar metallic behavior in both cases (albeit for different parameter values). In order to highlight the possibility of a correlation induced proximity to an unconventional superconducting state in undoped FeS, in this figure we also display the experimental resistivity data of tetragonal FeSe superconductor^63^. Compared to FeSe for *n* = 5.6 and *U* = 4.0 eV, with decreasing the on-site Coulomb interaction *ρ*_*dc*_(*T*) of metallic FeS becomes more incoherent-like below 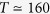 K, but the overall *T*-dependence resembles that of the FeSe system.

**Figure 7:**
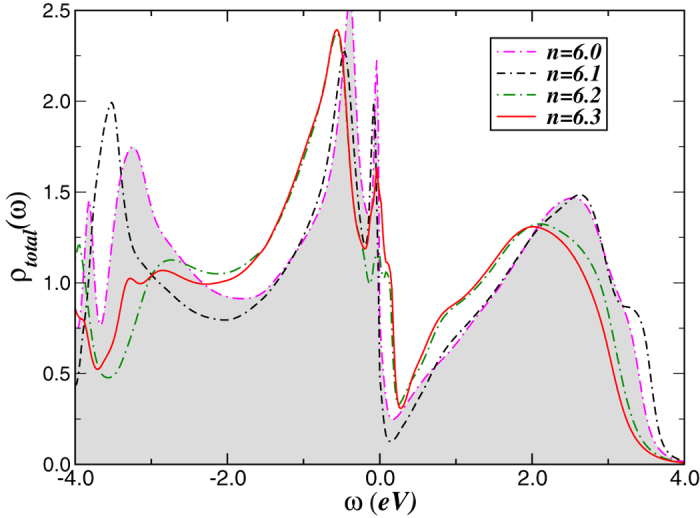
Total density-of-states (DOS) for the Fe 3*d* orbitals of stoichiometric and electron-doped FeS supercondutor with *U* = 3.6 eV and *J*_*H*_ = 0.7 eV. Notice the modification of the LDA+DMFT spectra due to large-scale transfer spectral weight induced by small changes in the Fe-3*d* total band filling, *n*. Selective microscopic coexistence of coherent-incoherent low-energy electronic states is predicted for electron-doped FeS supercondutor.

In Fig. 7 we show the changes in the correlated electronic structure upon electron doping (*n* > 6.0) metallic FeS parent compound. An intriguing observation is that a strong orbital reconstruction occurs at small electron doping. Hence, based on our previous LDA+DMFT results for iron-chalcogenide systems^40^, we shall attempt to predict features of the one-particle responses in the paramagnetic state of FeS superconductor. In Fig. 7, we show the total LDA+DMFT spectral functions. Clear changes in the pseudogaped features near *E*_*F*_ are visible for *n* = 6.0 and *n* = 6.1. Also interesting is low-energy electronic reconstruction near the Fermi energy with further increasing the averaged occupation number of the iron *d* shell, where a more quasicoherent normal state behavior sets in for electron-doping above *n* = 6.2. We propose that future photoemission and X-ray absorption spectroscopy results, which probe one-electron subtraction and addition spectra, can be directly compared with these: In particular, a broad incoherent peak below −0.4 eV should be seen in both pure and doped cases. Additionally, drastic modification of the LDA+DMFT spectra at high binding energies with *n* is visible, notwithstanding large-scale dynamical spectral weight transfer common in all cases. These are stringent tests for our proposal, and experimental verification should place it on solid ground.

## Conclusion

To summarize, we have used LDA+DMFT for a five-band Hubbard model to derive a correlation-induced orbital reconstruction in layered iron-chalcogenides. In particular, considering FeS as a suitable template, we have analyzed its paramagnetic insulating and metallic behavior, unraveling it as an effect of multi-orbital dynamical correlations. Also interesting is the orbital-selective electronic behavior obtained at the border of the Mott metal-insulator transition, where pseudogaped and Dirac fermions coexist at low energies. This coexistence arrises from charge-carrier scatterings due to interplay between multiband itinerance and electron-electron interactions, and this could be tested by a combination of spectral and transport measurements on metallic samples. Such studies are called for, and should confirm or refute our proposal of a marginal Dirac liquid for tetragonal FeS, which we ascribe to a lattice orthogonality catastrophe^64^ induced by orbital-selective Mottness. Whether novel quantum criticality associated with a selective-Mott transition in conjunction with the development of a marginal Dirac liquid will be seen in tetragonal FeS is in our view of great interest. Our work also provides a motivation to consider closer similarities between metallic FeS and the parent FeSe superconductor. As in FeSe, superconductivity in mackinawite manifests as an instability of an anomalous bad metal. Based on a theory-experiment comparison we suggest that mackinawite is ideal candidate for testing this and the idea of structural-induced electronic delocalization in the tetragonal phase of iron superconductors. Our findings thus provide a compelling motivation to study iron monosulfides and suggest a promising route to access Dirac fermion electronic reconstruction and unconventional superconductivity in non-Fermi liquid materials.

## Methods

To reveil insulating and metalic phases probed in electric transport experiments as well as the coexistence of pseudogaped and anisotropic Dirac-like electronic dispersion at the border of the Mott transition in tetragonal FeS, we employ the local-density-approximation plus dynamical-mean-field-theory (LDA+DMFT) which by construction takes into consideration the most relevant multi-orbital correlation effects and all-electron degrees of freedom. The state-of-the-art LDA+DMFT^60^ implementation used here also correctly describe disorder, pressure and temperature effects in multi-band electronic systems. The LDA+DMFT scheme is an ideal starting point towards the description of Coulomb-driven metal-to-insulator transitions, Fermi and non-Fermi liquid metallic states and in general grounds the role played by dynamical correlations in idealized many-particle models as well as in real multi-orbital systems^43^. The LDA+DMFT is a theoretical tool which provides realistic answers to interesting questions like why spin, orbital and magnetic orders in strongly correlated electron systems set in at low temperatures and how they might change upon application of external perturbations like pressure, chemical doping, magnetic and electric fields, etc. Here, the one-particle, LDA density-of-states are computed using the non-fully relativistic version of the PY-LMTO code^48^. To incorporate the effects of dynamical electronic correlations in this 3*d* transition-metal chalcogenide, we use the multi-orbital iterated-perturbation-theory (MO-IPT) as an impurity solver of the many-particle problem in DMFT, as described in detail in refs 50 and 51. Finally, we carried out the computation of electrical transport within the Kubo formalism^61^.

## Additional Information

**How to cite this article**: Craco, L. and Leoni, S. Selective orbital reconstruction in tetragonal FeS: A density functional dynamical mean-field theory study. *Sci. Rep.* **7**, 46439; doi: 10.1038/srep46439 (2017).

**Publisher's note:** Springer Nature remains neutral with regard to jurisdictional claims in published maps and institutional affiliations.
